# IGFBP-6 Alters Mesenchymal Stromal Cell Phenotype Driving Dasatinib Resistance in Chronic Myeloid Leukemia

**DOI:** 10.3390/life13020259

**Published:** 2023-01-17

**Authors:** Daniela Cambria, Lucia Longhitano, Enrico La Spina, Sebastiano Giallongo, Laura Orlando, Rosario Giuffrida, Daniele Tibullo, Paolo Fontana, Ignazio Barbagallo, Vincenzo Giuseppe Nicoletti, Giovanni Li Volti, Vittorio Del Fabro, Anna Rita Daniela Coda, Arcangelo Liso, Giuseppe Alberto Palumbo

**Affiliations:** 1 Division of Hematology, Department of General Surgery and Medical-Surgical Specialties, A.O.U. “Policlinico-Vittorio Emanuele”, University of Catania, 95123 Catania, Italy; 2 Department of Biomedical and Biotechnological Sciences, University of Catania, 95123 Catania, Italy; 3 Department of Medical Oncology, The Mediterranean Institute of Oncology, 95029 Viagrande, Italy; 4 Department of Medical and Surgical Sciences, University of Foggia, 71100 Foggia, Italy; 5 Department of Scienze Mediche Chirurgiche e Tecnologie Avanzate “G.F. Ingrassia”, University of Catania, 95123 Catania, Italy

**Keywords:** chronic myeloid leukemia, dasatinib, IGFBP6, TLR4, mesenchymal stromal cells

## Abstract

Chronic myeloid leukemia (CML), BCR-ABL1-positive, is classified as a myeloproliferative characterized by Philadelphia chromosome/translocation t(9;22) and proliferating granulocytes. Despite the clinical success of tyrosine kinase inhibitors (TKi) agents in the treatment of CML, most patients have minimal residual disease contained in the bone marrow microenvironment, within which stromal cells assume a pro-inflammatory phenotype that determines their transformation in cancer-associated fibroblasts (CAF) which, in turn can play a fundamental role in resistance to therapy. Insulin-like Growth Factor Binding Protein-6 (IGFBP-6) is expressed during tumor development, and is involved in immune-escape and inflammation as well, providing a potential additional target for CML therapy. Here, we aimed at investigating the role of IGFBP-6/SHH/TLR4 axis in TKi response. We used a CML cell line, LAMA84-s, and healthy bone marrow stromal cells, HS-5, in mono- or co-culture. The two cell lines were treated with Dasatinib and/or IGFBP-6, and the expression of inflammatory markers was tested by qRT-PCR; furthermore, expression of IGFBP-6, TLR4 and Gli1 were evaluated by Western blot analysis and immumocytochemistry. The results showed that both co-culture and Dasatinib exposure induce inflammation in stromal and cancer cells so that they modulate the expression of TLR4, and these effects were more marked following IGFBP-6 pre-treatment suggesting that this molecule may confer resistance through the inflammatory processes. This phenomenon was coupled with sonic hedgehog (SHH) signaling. Indeed, our data also demonstrate that HS-5 treatment with PMO (an inducer of SHH) induces significant modulation of TLR4 and overexpression of IGFPB-6 suggesting that the two pathways are interconnected with each other and with the TLR-4 pathway. Finally, we demonstrated that pretreatment with IGFBP-6 and/or PMO restored LAMA-84 cell viability after treatment with Dasatinib, suggesting that both IGFBP-6 and SHH are involved in the resistance mechanisms induced by the modulation of TLR-4, thus indicating that the two pathways may be considered as potential therapeutic targets.

## 1. Introduction

Chronic Myeloid Leukemia (CML) is a myeloproliferative disorder that originates in the hematopoietic stem cell (HSC) compartment of the bone marrow and characterized by the presence of the Philadelphia chromosome (Ph chromosome), rising by a translocation between chromosome 9 and chromosome 22, 22 and resulting in a chimeric gene that codes for a protein with a constitutive tyrosine kinase activity [1,2,3,4,5]. The amino-terminal portion of the ABL protein contains the SRC homology regions: SH1, SH2 and SH3. The SH1 domain has catalytic activity, the SH2 domain has phosphotyrosine binding activity, whereas the activity of the ABL protein is negatively regulated by the SH3 domain, the deletion of which involves the transformation of ABL into an oncogene [5].

The BCR-ABL fusion protein is mainly involved in several cytoplasmatic signaling pathways including MAPK (Ras-mitogen-activated protein kinase), JAK-STAT (Janus-activated kinase) and PI3K/AKT (phosphoinositide 3-kinase) [6,7].

The development of BCR-ABL tyrosine kinase inhibitors (TKIs) has revolutionized the therapy for CML. TKIs are highly effective in inducing remission, preventing disease progression and prolonging patient survival during the chronic phase. However, treatment with TKIs has some limitations such as the failure of therapy in a small subgroup of patients resistant to this therapy, that show the persistence of leukemic stem cells (CSL) [8,9].

The tumor microenvironment (TME) is affected by the presence of various components that form a dynamic complex including, the extracellular matrix (ECM), stromal cells, fibroblasts, mesenchymal cells, pericytes, occasionally adipocytes, lymphatic and blood vascular networks and immunity cells such as T and B lymphocytes, natural killer cells and tumor-associated macrophages (TAMs). The interaction of these elements plays an active role in the promotion of cancer progression. It is, indeed, well known that the increased oxidative stress and acidosis observed in this context are associated with tissue dysplasia This interaction increases the oxidative stress and acidosis and so leading to dysplasia [10,11,12,13,14,15,16].

The medullary niche is the best-characterized tumor microenvironment of the bone marrow, indeed is involved in the regulation of stem cells hematopoiesis [17,18]. In CML, the BCR-ABL protein, with constitutive tyrosine kinase activity, transforms hematopoietic stem cells or hematopoietic progenitor cells into CSLs, with self-renewal, proliferation, and differentiation activities under the influence of signals from the bone marrow microenvironment. This crosstalk can make the stromal cells assume a pro-inflammatory phenotype that determines their transformation into tumor-associated fibroblasts (CAF) that play a fundamental role in the onset of resistance to therapy [19].

The IGF system is crucial during cell growth [20]. This system includes different protein complexes such as IGF-I and IGF-II, and their high affinity binding proteins (IGFBP from 1 to 6). IGFBPs exert their mitogenic actions mainly through activation of the IGF receptor type 1 (IGF-1R); this interaction leads to the activation of pathways associated with proliferation, including AKT and ERK which contribute to resistance to anticancer therapies [21,22,23]. Among all IGFBP binding molecules studied, IGFBP-6 inhibits IGF-II and it has activities such as proliferation, differentiation, migration, and cell survival; but it has little or no effect on the action of IGF-I, in part due to the low affinity of IGFBP-6 for this ligand [24,25,26]. 

The sonic hedgehog (SHH) signaling pathway is another fundamental player in the normal development of invertebrates and vertebrates [27,28]. SHH is involved in the maintenance of somatic stem cells and pluripotent cells, which are critical process for either tissue repair or tumorigenesis [29,30,31,32,33,34,35,36].

Activation of SHH signaling involves three proteins: SHH, Patched (PTCH) and Smoothened (SMO) [37]. The canonical signaling pathway begins with the binding of SHH to its cognate receptor Patched 1 transmembrane protein (PTCH1). The activity of SMO is constitutively repressed by PTCH1, and this inhibition is released by the binding of SHH to PTCH1 [27].

Toll-like receptors belong to the TLR-IL-1 receptor (TIR) superfamily; they have an external domain consisting in the recognition of pathogen-associated molecular patterns (PAMPs) and an intracytoplasmic TIR domain that mediates the recruitment of adapter molecules. Most TLRs are expressed on the cell surface and bind lipids and proteins (TLR1, 2, 4, 5, 6), while TLR3, 7, 8 and 9 are found at the endosome level where they are activated following capture and internalization of pathogens and their products [38,39]. We recently demonstrated that IGFBP-6/SHH/TLR4 axis is implicated in alterations of the primary myelofibrosis microenvironment and that IGFBP-6 may play a central role in activating SHH pathway during the fibrotic process [40]. In this work we highlighted the role of IGFBP-6/SHH/TLR4 axis in TKi response. 

## 2. Materials and Methods

### 2.1. Cell Lines

The cell lines used for the experiments are LAMA84-s (ATCC CRL-3347 ™) from chronic myeloid leukemia in the blast phase and HS-5 (ATCC CRL-11882 ™) healthy bone marrow stromal cells. The LAMA-84 were grown in RPMI 1640 medium in which 10% FBS and 1% penicillin / streptomycin were added, instead the HS-5 were grown in DMEM medium also enriched with 10% FBS. and 1% penicillin/streptomycin. Co-cultures were carried out in a 1:5 ratio (medium 1:5 DMEM high glucose/RPMI 1640) between HS-5 and LAMA-84. After 24 h of treatment non-adherent cells (LAMA-84) were removed, whereas HS5 were selected by their adherence to the plastic plates. Flow cytometry analysis was performed for the evaluation of the purity of the cell (CD45+ cells for the LAMA84 and CD45- for the HS5). Both cell lines were incubated at 37 °C in a humid atmosphere with 5% CO_2_ [41,42,43]. The experiments were performed on both single cell lines and co-cultures of stromal cells and chronic myeloid leukemia cells. 

### 2.2. XTT Assay

XTT (Sigma) is a colorimetric assay based on the reduction of 2,3-bis (2-methoxy-4-nitro-5-Sulfophenyl)-5-[(phenylamino) carbonyl]-2Htetrazolium ydroxide) in formazan, a water-soluble compound of orange color directly quantifiable by the spectrophotometer. The reduction of XTT occurs inside the cell and is operated by enzymes, therefore it requires metabolically active cells. Cells were seeded in 96-well plates (2 × 10^4^ cells/mL HS-5; 1 × 10^5^ cells/mL LAMA84) and incubated using dasatinib at a concentration of 1 nM for 48 hrs on both monoculture and co-culture cell lines. Furthermore, cell viability was also measured after pretreatment with IGFBP-6 or purmorphamine (a smoothened (Smo) receptor agonist and activates the Hedgehog pathway, PMO) 1 µM for 24 hrs and subsequent treatment with dasatinib 1 nM for further 48 hrs. At the end of the incubation, 25 µL/well of XTT were added and incubated for 4 hrs. Subsequently, the absorbance was measured at 450 nm (reference wavelength) with a Multiskan SkyHigh Microplate Specrophotometer plate reader (Thermo Fisher Scientific, Milan, Italy).

### 2.3. Western Blot Analysis

Western blot analysis was performed on HS-5, LAMA-84 in monoculture and on single cell lines that were co-cultured with each other. The cells were lysed in cold saline phosphate buffer containing 1% of Triton X-100. The total protein content was quantized and an equal amount of 50 μg protein for each sample is denatured for 5 min in Laemmli’s buffer. The separation of proteins was performed by electrophoresis using a 12% polyacrylamide gel (Mini Protean II System, Bio-Rad, Herts, UK) followed by the electro-transfer of proteins onto the nitrocellulose membrane. Subsequently, the membranes were blocked using Odyssey Blocking Buffer (Licor, Milan, Italy) for one hour at room temperature. After blocking the nonspecific sites, the membranes were washed three times with phosphate saline buffer (PBS) for 5 min and incubated overnight at 4 °C with the following primary antibodies resuspended in Odyssey Blocking Buffer: anti-IGFBP-6 (1:500, abcam), anti-β-Actin (1:5000, abcam), anti-α-SMA (1:500, abcam). After incubation the membranes were washed three times with 0.1% PBS with Tween for 5 min and subsequently incubated with secondary infrared anti-mouse (Alexa Fluor 488) and anti-rabbit (Alexa Fluor 620) antibodies resuspended in Odyssey Blocking Buffer for one hour. The bands were visualized using the Odyssey Infrared Imaging Scanner instrument (Licor, Milan, Italy) and the protein levels were quantified by densitometric analysis using the ImageJ software. Data were normalized on β-actin expression levels [44,45,46].

### 2.4. Immunofluorescence

Immunofluorescence was performed on cells previously fixed in 4% paraformaldehyde, permeated with 0.1% Triton 100X and incubated in block solution (10% normal goat serum, NGS, 0.1% Triton 100X in PBS) for one hour at room temperature [47,48,49]. The samples were incubated overnight at 4 °C with the following antibodies diluted in PBS: anti-IGFBP-6 (1:100, Abcam). After incubation, the samples were washed and incubated for one hour at room temperature with the specific secondary fluorescent antibody for each primary antibody used. The nuclei were labeled with 4′,6-diamidino-2-phenylindole (Dapi, 1:1000, Cat#: D1306, Invitrogen) for 5 min at room temperature. The slides were mounted using the Permafluor fluorescent mounting medium (ThermoScientific) and the digital images were acquired using the Leica DM IRB fluorescence microscope or the Leica TCS SP8 confocal microscope (Leica Microsystems, Buccinasco, Milano, Italy).

### 2.5. RT-PCR Analysis

Cellular RNA was extracted according to the Trizol protocol (Thermofisher scientific) [50,51,52]. The cDNA was synthesized by reverse transcription of 1 µg of total RNA using the Applied Biosystem reverse transcription kit (Foster City, CA, USA). The expression levels of IGFBP-6, SIRT1, PGC1a, TGF-β, IFNg, SHH, TLR-4 were evaluated (Table 1). For each sample, gene expression levels were normalized using β-actin expression levels.

### 2.6. Statistical Analysis

Statistical analysis was performed using Prism Software using two-tailed unpaired Student’s t test for comparison of *n* = 2 groups. Comparisons of *n* > 2 groups were performed using a one-way ANOVA and Holm-Sidak’s multiple comparisons test. (Graphpad Software Inc., California, USA, RRID: rid_000081). Data are expressed as mean ± SD, unless otherwise stated. For all statistical tests, *p* values < 0.05 were considered statistically significant.

## 3. Results

### 3.1. Dasatinib Exposure Does Not Affect LAMA-84 Cell Viability in Coculture with Human Mesenchymal Stem Cells (HS-5)

**Figure 1:**
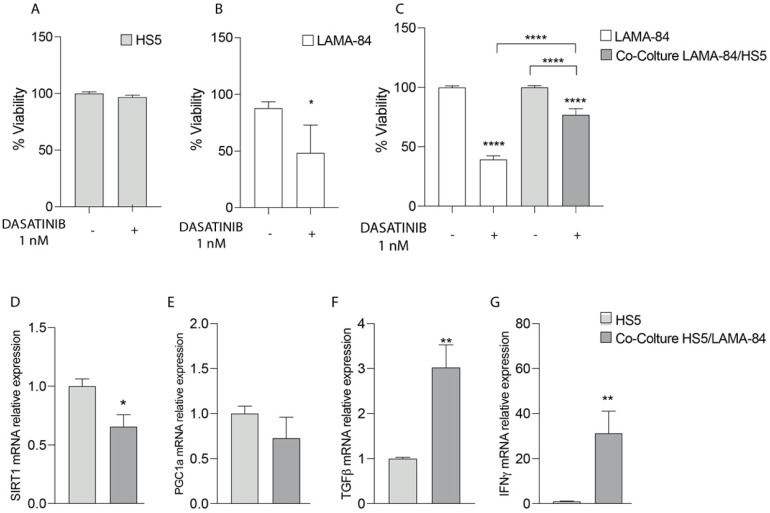
Cell viability of HS−5 (**A**), LAMA−84 (**B**) and LAMA−84 in coculture with HS−5 (**C**) after treatment with 1 nM dasatinib. Relative expression of SIRT-1 (**D**), PGC1a (**E**), TGF-β (**F**) and INFγ (**G**) in HS−5 in coculture with LAMA−84 compared to HS−5 in monoculture. * *p* < 0.1; ** *p* < 0.01; **** *p* < 0.0001.

To test in vitro sensitivity to dasatinib, LAMA-84 alone or in co-culture with HS-5 cells were treated at 1 nM for 48 hrs and cell viability by using XTT assay was measured. In particular, as shown in Figure 1A,B, the HS-5 cell proliferation rate was not affected after dasatinib exposure compared to LAMA-84 alone (Figure 1A,B). On the contrary, by performing XTT in LAMA-84 cocultured with HS-5, decreased cell viability appeared in LAMA-84 cells (Figure 1C). More specifically, to evaluate which molecular pathway allows HS-5 to be less sensitive to treatment and to investigate in depth the phenotype into which they may be polarized alone or in coculture with LAMA-84, levels of in the SIRT1, PGC1α, TGF-β and IFN-γ gene expression profile were measured. Surprisingly, the downregulation of SIRT1 associated with the decreasing PGC1α trend (Figure 1D,E), suggests a potential decrease in mitochondrial biogenesis also associated with a metabolic switch towards a more glycolytic one. Moreover, the increase in TGF-β and IFN-γ levels confirmed HS-5 polarization toward a pro- inflammatory CAF phenotype (Figure 1F,G).

### 3.2. Dasatinib Increases IGFBP-6 Levels in Coculture Conditions and Pre-Treatment with IGFBP-6 Leads to Increased Cell Viability in LAMA-84 Cells

**Figure 2:**
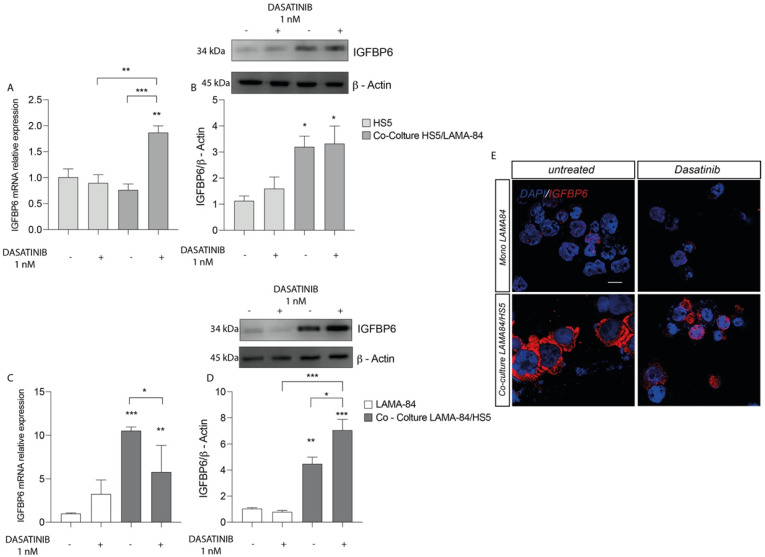
Relative expression of IGFBP6 transcript (**A**) and protein (**B**) in HS−5 cells coculture with LAMA−84 versus HS−5 monoculture after treatment with dasatinib 1 nM. Relative expression of IGFBP6 transcript (**C**) and related protein (**D**) in LAMA−84 in coculture with HS−5 compared to LAMA−84 in monoculture after treatment with dasatinib 1 nM. (**E**) Immunofluorescence analysis of IGFBP6 (IGFBP6 in red, nucleus in blue) in LAMA−84 in coculture with HS−5 compared to LAMA-84 in monoculture after treatment with dasatinib. * *p* < 0.05; ** *p* < 0.01; *** *p* < 0.001.

We first assessed the effects in HS-5 cells of dasatinib treatment. We finding an IGFBP-6 mRNA expression levels increase in HS-5 previously cocultured with LAMA-84 compared to HS-5 alone and untreated cocultures (Figure 2A). Following this finding, western blot analysis showed increased IGFBP-6 protein expression levels in HS-5 previously cocultured with LAMA-84 (Figure 2B). Similarly, the IGFBP-6 mRNA expression profile in LAMA-84 cells was increased after coculture with HS-5 (Figure 2C). Regarding LAMA-84, it was demonstrated a striking increase in IGFBP-6 protein expression when in coculture with HS-5 after dasatinib treatment (Figure 2D). By performing immunofluorescence analysis, it was confirmed that IGFBP-6 levels were higher in LAMA-84 in coculture with HS-5 than in LAMA-84 alone (Figure 2E). 

### 3.3. Pre-Treatment with IGFBP-6 Increases SHH Expression Levels in Coculture Models and Activates TLR-4 Signalling

**Figure 3:**
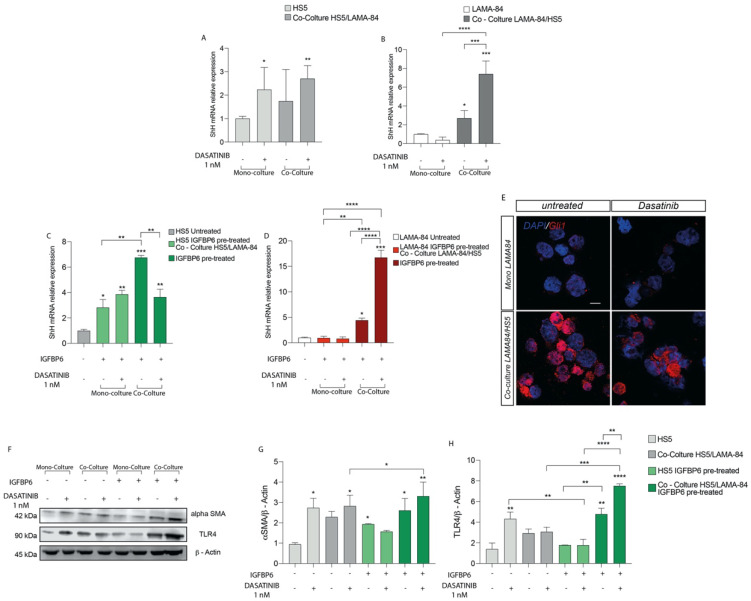
Relative expression of SHH transcript in HS−5 (**A**) and LAMA−84 (**B**) in coculture, with or without 1 nM dasatinib. Relative expression of SHH transcript in HS−5 (**C**) and LAMA−84 (**D**) in coculture, with or without IGFBP6 and dasatinib 1 nM. Immunofluorescence analysis of the expression of Gli1 in LAMA−84 in coculture with HS−5 in the presence or not of dasatinib 1 nM (**E**). Relative expression of aSMA and TLR4 protein in HS−5 in coculture with LAMA−84 with or without pretreatment with IGFBP6 compared to HS−5 in monoculture with or without pretreatment with IGFBP6 (**F**–**H**) (* *p* < 0.1; ** *p* < 0.01; *** *p* < 0.001; **** *p* < 0.0001).

As shown in Figure 3A, after dasatinib exposure, SHH mRNA expression was upregulated both in HS-5 alone or in coculture with LAMA-84 and in LAMA-84 previously cocultured with HS-5. These data were further confirmed measuring by qRT-PCR a SHH mRNA upregulation in LAMA-84 cocultured with HS-5 (Figure 3B). To assess whether the combination IGFBP-6-dasatinib had an additive effect on SHH expression, both HS-5 and LAMA-84 monocultures and HS-5 cocultured with LAMA-84 were pre-treated with IGFBP-6 followed by dasatinib. This treatment led to an increase in SHH gene expression levels, markedly in HS-5 previously cultured with LAMA-84 (Figure 3C), as well as in LAMA-84 previously cultured with HS-5, especially after dasatinib exposure (Figure 3D). By performing immunofluorescence analysis in LAMA-84 alone or cocultured with HS-5, it was confirmed the SHH pathway activation. Indeed, Gli1 levels in LAMA-84 previously cultured with HS-5 were increased compared to LAMA-84 alone (Figure 3E). Furthermore, to evaluate whether dasatinib alone or in combination with IGFBP-6 may influence HS-5 phenotype, alone or in coculture with LAMA-84, western blot analysis of α-SMA protein expression was performed in the coculture models. As a demonstration of cell conditioning, the α-SMA protein raised expression levels under both conditions, especially in HS-5 previously cocultured with LAMA-84 pre-treated with IGFBP-6 and later with dasatinib (Figure 3F,G). Surprisingly, TLR-4 protein expression was upregulated in HS-5 previously cultured with LAMA-84, pre-treated with IGFBP-6 and later exposed to dasatinib. Moreover, significantly increased TLR-4 protein levels were found in HS-5 previously cocultured with LAMA-84 and treated with only IGFBP-6. Finally, TLR-4 overexpression was also found in HS-5 cocultured with LAMA-84 with or without IGFBP-6 pre-treatment while TLR-4 downregulation was found in HS-5 pre-treated with IGFBP-6 and subsequently with dasatinib, compared to HS-5 alone treated with dasatinib only (Figure 3F–H).

### 3.4. The IGFBP-6-Dasatinib Combination Treatment Increases HS-5 Inflammatory State 

**Figure 4:**
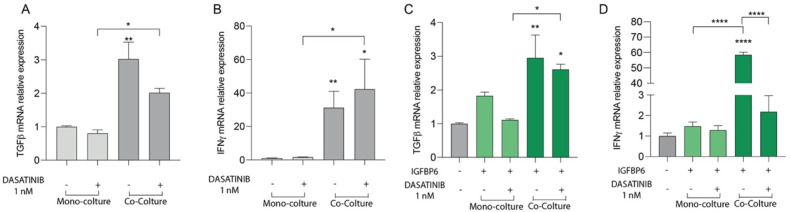
Relative expression of TGF−β (**A**) and INFγ (**B**) transcript in HS−5 coculture with LAMA−84, with or without 1 nM dasatinib. Relative expression of TGF−β (**C**) and INFγ (**D**) transcript in HS−5 in coculture with LAMA−84, with or without IGFBP6 and dasatinib 1 nM. (* *p* < 0.1; ** *p* < 0.01; **** *p* < 0.0001).

To evaluate the effect of dasatinib alone or in combination with IGFBP-6 on mesenchymal stromal cells inflammatory state, TGF-β and IFN-γ mRNA expression levels were evaluated. After 48 h of dasatinib exposure, HS-5 in coculture with LAMA-84 showed TGF-β and IFN-γ mRNA upregulation compared to HS-5 alone (Figure 4A,B). Furthermore, HS-5 previously cocultured with LAMA-84 and pre-treated with IGFBP-6 showed TGF-β and IFN-γ mRNA increase compared to HS-5 in monoculture (Figure 4C,D). 

### 3.5. The IGFBP-6 + Dasatinib Combination Treatment Increases LAMA-84 Inflammatory State and Activates TLR-4 Signalling

**Figure 5:**
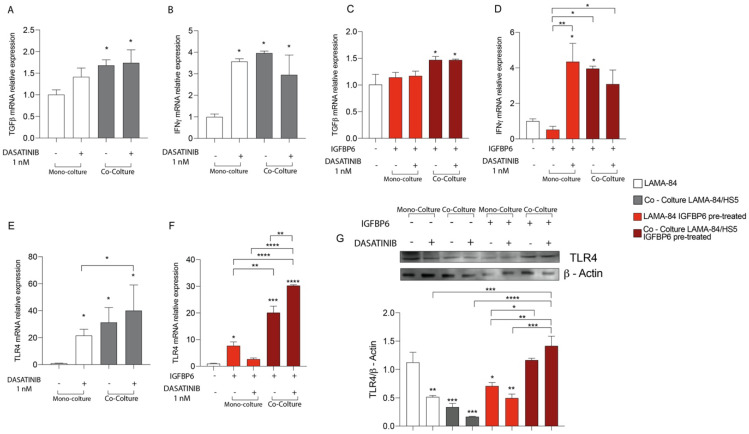
Relative expression of TGF−β (**A**) and INFγ (**B**) transcript in LAMA−84 coculture with HS-5, with or without 1 nM dasatinib. Relative expression of TGF−β (**C**) and INFγ (**D**) transcript in LAMA−84 coculture with HS−5, with or without IGFBP6 and dasatinib 1 nM. Relative expression of TLR4 transcript in LAMA−84 in coculture with HS-5, with or without dasatinib 1 nM (**E**). Relative expression of TLR4 transcript in LAMA−84 in coculture with HS−5, with or without IGFBP6 and dasatinib 1 nM (**F**). Relative expression of TLR4 protein in LAMA−84 in monoculture and in coculture with HS−5, with or without IGFBP6 and dasatinib 1 nM (**G**). * *p* < 0.05; ** *p* < 0.01; *** *p* < 0.001; **** *p* < 0.0001.

To evaluate whether dasatinib may also affect the LAMA-84 inflammatory state as described for HS-5 (see above), LAMA-84 alone or in coculture with HS-5 were treated with dasatinib at 1 nM for 48 hrs. After this time, TGF-β and IFN-γ mRNA expression levels were measured and while TGF-β was significantly increased in LAMA-84 cocultured with HS-5, IFN-γ levels increased in both LAMA-84 alone and in coculture with HS-5 after dasatinib treatment (Figure 5A,B). As shown in Figure 5C, TGF-β levels were also increased in LAMA-84 in coculture with HS-5 and IGFBP-6 pre-treated compared to LAMA-84 alone without IGFBP-6 pre-treatment. Furthermore, IGFBP-6 + dasatinib combination treatment results in a significant increase in IFN-γ levels in LAMA-84 alone compared to LAMA-84 treated with IGFBP-6 only (Figure 5D). In addition, TLR-4 mRNA expression levels were higher in LAMA-84 in coculture with HS-5 treated with dasatinib than in both LAMA-84 alone treated or not with dasatinib (Figure 5E). IGFBP-6 pre-treatment subsequent to dasatinib treatment led to increased TLR-4 expression levels in LAMA-84 cocultured with HS-5 compared to LAMA-84 in co-culture treated with IGFBP-6 only. Moreover, increased TLR-4 mRNA levels were observed in LAMA-84 monoculture pre-treated with IGFBP-6 (Figure 5F). Finally, these data were confirmed by western blot analysis, showing that TLR-4 protein expression was higher both in LAMA-84 previously cocultured with HS-5 treated with IGFBP-6 in combination or not with dasatinib compared to LAMA-84 alone or untreated cocultures (Figure 5G).

### 3.6. IGFBP-6 Rescues LAMA-84 Cell Viability after Dasatinib Exposure

**Figure 6:**
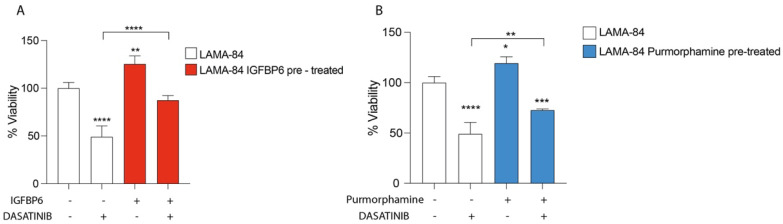
LAMA−84 Cell Viability after IGFBP6 (**A**) and PMO, Hedgehog pathway activator (**B**) pre-treatment. * *p* < 0.05; ** *p* < 0.01; *** *p* < 0.001; **** *p* < 0.0001.

To evaluate the impact of IGFBP-6 and PMO on LAMA-84 cell viability, cell cultures were pre-treated with both molecules and later treated with dasatinib. Interestingly, LAMA-84 showed an increase in cell viability compared to LAMA-84 treated with dasatinib alone, conferring a protective role of IGFBP-6 and PMO against dasatinib exposure (Figure 6).

## 4. Discussion

The bone marrow microenvironment is a highly complex tissue, containing many cell-type such as mesenchymal stromal cells, osteoblasts, osteoclasts, endothelial cells, and neural cells interacting with healthy hematopoietic stem cells through different molecules and signalling pathways [30,31,32,53,54,55]. As reported by previous studies, drug resistance and disease progression are deeply related to the tumor microenvironment and more specifically to the stromal counterpart [56,57]. Our experiments demonstrated that cells HS-5 are not affected by dasatinib treatment as compared to LAMA-84, which, in turn, show higher sensitivity when in monoculture than in co-culture with HS-5, confirming that the stroma could play a key role in CML dasatinib resistance. The cross-talk between CML-LSCs and the bone marrow microenvironment is mediated by several molecules via both paracrine and autocrine mechanisms. Among these molecules, a fundamental role in tumor progression is played by IGFBPs. Several studies showed that IGFBPs can bind their specific receptors and activate signalling pathways which, in turn, modulate several cellular processes [58].

Since our data showed that co-culture between HS-5 and LAMA-84 leads to an increase in the IGFBP-6 both mRNA and protein expression levels and that LAMA-84 cell viability was higher after IGFBP-6 and dasatinib treatment than in LAMA-84 with dasatinib alone, we propose that IGFBP-6 could be involved in CML cell lines’ resistance mechanisms rescuing them from apoptosis. However, future studies will be needed to confirm our data in other CML models. It is well known that, in several tumor types, progression and resistance to the most common therapies are due to the pivotal role played by CAFs [59], which in turn promote cancer cells growth and invasion through extracellular matrix soluble factors secretion, tumor cells physical interactions, angiogenesis regulation, immunity and metabolism regulation as well. Treatment with IGFBP-6 determines the development of a pro-inflammatory CAF phenotype in HS-5 as demonstrated by α-SMA, FAP1 and TGF-β protein levels increase and MMP9, MMP2, CHI3L1, and TIMP2 release into the extracellular environment. In the last few years, Sonic-Hedgehog (SHH) signaling has been recognized as an important pathway for promoting tumor progression and by now it is widely recognized as a therapeutic target [60,61]. In Mammals, SHH pathway transcription factors are associated with glioma-associated oncogene (Gli), and have three homologs, Gli1, Gli2, and Gli3; in particular, Gli1 target genes identification allowed us to understand the involvement of this gene in the proliferation, apoptosis, cell adhesion, angiogenesis processes, and metastatic potential as well [27,62,63].

As previously reported, CML cells show increased SHH signaling pathways players, making them ideal candidates for leukemia development [64]. Since SHH inhibition doesn’t affect physiological hematopoietic stem cells and can inhibits leukemic stem cells, exposure of CML leukemic stem cells to cyclopamine (SHH signaling inhibitor, CYC) reduces their number and inhibits their growth [65,66,67].

The experiments described here, allowed us to demonstrate that IGFBP-6 trigger SHH activation in HS-5, as suggested by the increase in Gli1 levels; moreover IGFBP-6 and SHH in HS-5 lead to α-SMA and TGF-β increase with the subsequent MMP9, CHI3L1 and TIMP2 release into the extracellular environment, confirming that both molecules are involved in the transformation processes of stromal cells into CAF with specific pro-inflammatory phenotype. The data obtained so far, allowed us to speculate that both IGFBP-6 and SHH determine HS-5 transformation into CAF and that combining dasatinib with IGFBP-6, α-SMA levels in coculture increase and this is more marked only in HS-5 previously co-cultured with LAMA-84 and pre-treated with IGFBP-6, this in turn suggesting the latter contributes substantially to the activation of the CAF phenotype. Furthermore, increased SHH levels in HS-5 are due to both co-cultures and dasatinib treatment. Interestingly, dasatinib treatment of HS-5 previously co-cultured with LAMA-84 and treated with IGFBP-6 showed higher SHH levels suggesting intimate relation between these two pathways. Similarly, also LAMA-84 previously co-cultured with HS-5 showed higher SHH levels after dasatinib treatment and this increase is highlighted by IGFBP-6 pre-treatment and later with dasatinib. Notably, co-culture without any other treatment increases the inflammatory state, and it may well contribute to creating an optimal environment promoting cancer progression. Since inflammation is one of the factors involved in the tumorigenesis process, and TLR-4 represents the most studied pathway, several authors have shown that TLR-4 activation leads to IL-8 and IL-6 increased expression in breast cancer, also associated with VEGF and TGF-β increased expression in prostate cancer, in which their overexpression is also very often associated with poor prognosis [68].

## 5. Conclusions

**Figure 7:**
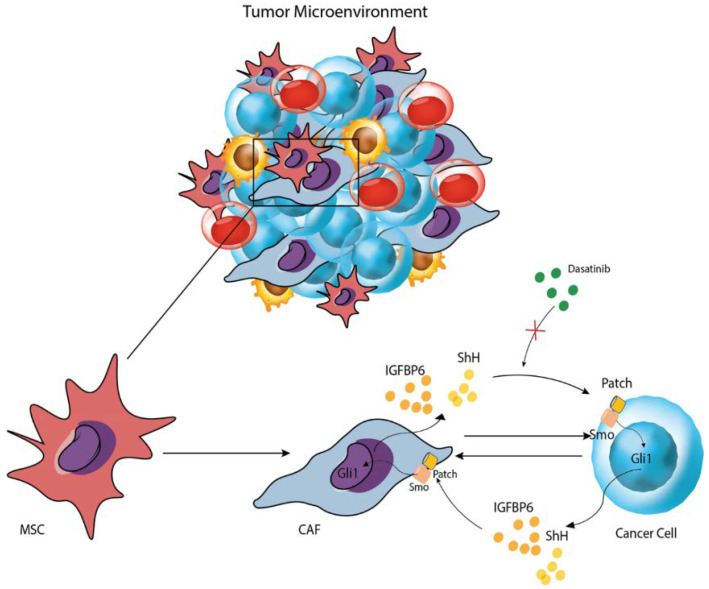
Graphical representation of the mechanism proposed.

This study shows that both co-culture and dasatinib administration induce an inflammatory state in both HS-5 and LAMA-84 cell lines, as demonstrated by TGF-β and IFN-γ increase. Moreover, IGFBP-6 pre-treatment augments this phenomenon suggesting that IGFBP-6 may confer resistance through the inflammatory processes activation as confirmed by TLR-4 modulation in both HS-5 and LAMA-84 after IGFBP-6 and dasatinib treatment (Figure 7). The same TLR-4 modulation was observed after HS-5 treatment with PMO, in combination or not with CYC, suggesting that SHH directly modulates TLR-4. Our data also demonstrate that HS-5 treatment with PMO induces a significant overexpression of IGFPB-6. These findings, taken together with those previously reported that HS-5 treatment with IGFBP-6 induces an increase in SHH demonstrate that SHH and IGFBP-6 modulate each other. In addition, IGFBP-6 is involved in the modulation of TLR-4, as demonstrated after HS-5 treatment with IGFBP-6, CYC, and their combination, thus suggesting that the two pathways are interconnected with each other and with the TLR-4 pathway [40]. Finally, IGFBP-6 and /or PMO pre-treatment, resulting in a rescue of LAMA-84 cell viability after dasatinib treatment compared to those treated with dasatinib alone, may suggest that both IGFBP-6 and SHH are involved in resistance mechanisms induced by TLR-4 modulation. In conclusion, IGFBP-6 and SHH pathways may be considered as potential candidates for therapeutic interventions and could lead to new targeted strategies, aiming to overcome resistance to TKi, although the underlying resistance mechanisms to these drugs are not yet fully understood and require further investigations.

## Figures and Tables

**Table 1:** Primers.

Gene of Interest	Forward Primer (5′ → 3′)	Reverse Primer (5′ → 3′)
SIRT1	AGGCCACGGATAGGTCCATA	GTGGAGGTATTGTTTCCGGC
PGC1α	ATGAAGGGTACTTTTCTGCCCC	GGTCTTCACCAACCAGAGCA
TGF-β	CCCAGCATCTGCAAAGCTC	GTCAATGTACAGCTGCCGCA
IFNγ	TGAATGTCCAACGCAAAGCA	CGACCTCGAAACAGCATCTGA
IGFBP-6	CCTGCTGTTGCAGAGGAGAAT	CTCTGCGGTTCACATCCTGT
SHH	GCGAGATGTCTGCTGCTAGT	TTACACCTCTGAGTCATCAGC
TLR4	AAGCCGAAAGGTGATTGTTG	CTGAGCAGGGTCTTCTCCAC
βActin	CCTTTGCCGATCCGCCG	AACATGATCTGGGTCATCTTCTCGC

## Data Availability

The data presented in this study are available in the article.
